# Resumption of migraine preventive treatment with CGRP(-receptor) antibodies after a 3-month drug holiday: a real-world experience

**DOI:** 10.1186/s10194-022-01417-9

**Published:** 2022-03-30

**Authors:** Bianca Raffaelli, Maria Terhart, Jasper Mecklenburg, Lars Neeb, Lucas Hendrik Overeem, Anke Siebert, Maureen Steinicke, Uwe Reuter

**Affiliations:** 1 Department of Neurology, Charité Universitätsmedizin Berlin, Charitéplatz 1, 10117 Berlin, Germany; 2 Clinician Scientist Program, Berlin Institute of Health at Charité (BIH), Berlin, Germany; 3 Universitätsmedizin Greifswald, Greifswald, Germany

**Keywords:** Migraine, CGRP, Antibodies, Discontinuation, Resumption

## Abstract

**Background:**

Migraine frequency increases after the cessation of successful preventive treatment with CGRP(-receptor) monoclonal antibodies (mAbs). In this study, we aimed to evaluate the course of migraine after treatment resumption.

**Methods:**

Patients with migraine, who started treatment with the same CGRP(-R) mAb after a three-month drug holiday were included in this analysis. We collected headache data at four prospective visits: 1) during the four weeks before the initial mAb treatment (baseline); 2) during the four weeks before the last mAb injection; 3) in weeks 13–16 of the drug holiday; 4) in weeks 9–12 after treatment restart. Outcomes were the changes in monthly migraine days (MMD), monthly headache days (MHD), monthly days with acute medication use (AMD) and Headache Impact Test-6 (HIT-6) scores across the observation period.

**Results:**

This study included 39 patients (erenumab *n* = 16; galcanezumab/ fremanezumab *n* = 23). MMD decreased from 12.3 ± 6.3 at the end of the drug holiday to 7.8 ± 5.5 three months after treatment restart (*p* = 0.001). The improvement after treatment resumption was similar to the response in the initial treatment period (baseline: 12.3 ± 6.3 MMD vs. 7.5 ± 5.2 MMD before treatment interruption). MHD and AMD showed a significant improvement after treatment restart. HIT-6 scores decreased, indicating a diminished impact of headache on everyday life.

**Conclusions:**

Reinitiation of treatment with CGRP(-R) mAbs after a drug holiday leads to a significant reduction of migraine frequency and medication use as well as improvement in quality of life.

## Introduction

Three monoclonal antibodies (mAbs) targeting the Calcitonin Gene-Related Peptide (CGRP) pathway are currently available in Germany as specific migraine preventive treatments: erenumab binds to the CGRP-receptor (CGRP-R), while galcanezumab and fremanezumab target CGRP directly [1].

The efficacy and tolerability of CGRP(-R) mAbs have been demonstrated in large trial programs and confirmed in numerous real-world studies [2–13]. However, several questions remain open in the management of these novel drugs.

One key issue is the optimal treatment duration and the need for treatment discontinuation after a period of successful therapy. The expert consensus of the European Headache Federation (EHF) recommends a discontinuation attempt after 6–12 months to reevaluate the need for preventive therapy [14]. Current real-world studies have shown a progressive worsening of migraine frequency during such a discontinuation attempt [15–17]. In most patients, migraine frequency rapidly returned to the levels before the start of prophylactic mAb therapy [15–17].

Based on our experience, approximately 90% of patients resume treatment within three months after discontinuation [17]. However, the course of migraine after mAb therapy reinitiation remains unknown.

From a clinical point of view, treatment resumption would ideally lead to an improvement of migraine frequency similar to the initial treatment. A predictable good response after restart would facilitate the decision to temporarily stop treatment and begin again in case of disease deterioration. Alternatively, a second treatment cycle after a long pause could potentially be less effective than the first one, for example due to habituation effects. The evaluation of headache parameters during a second treatment cycle is therefore a crucial step towards improved care of patients with migraine. We have recently published data on migraine frequency and quality of life after mAbs discontinuation [17]. We now report migraine characteristics following the reinitiaton of CGRP(-R) mAb therapy after a three-month discontinuation attempt.

## Methods

### Study design and participants

We conducted a longitudinal cohort study at the Headache Center of the Charité – Universitätsmedizin Berlin.

Eligible patients were selected from a previous study cohort [17]. All patients had a diagnosis of episodic or chronic migraine according to the International Classification of Headache Disorders 3 (ICHD-3) criteria. The subjects of this study received preventive treatment with CGRP(-R) mAbs and underwent a discontinuation attempt after at least 8 months of therapy according to the EHF treatment guidelines for the prophylaxis of migraine with mAbs [14]. All included patients reported good tolerability of mAb treatment during the first treatment cycle [17]. The diagnosis of episodic or chronic migraine was based on the migraine characteristics in the year before the initial start of CGRP(-R) mAbs.

Inclusion criteria for this analysis were:

- restart of preventive treatment with CGRP(-R) mAbs after three months of treatment interruption
- treatment with the same CGRP(-R) mAb as in the first treatment cycle
- complete headache documentation for three months after treatment restart.

The start of a concomitant migraine preventive treatment led to exclusion from the analysis.

Depending on the mAb they received patients were divided in a CGRP-R mAb group (i.e. treatment with erenumab 140 mg s.c. per month) and a CGRP-ligand mAb group (i.e. treatment with galcanezumab 120 mg s.c. per month after a 240 mg loading dose or fremanezumab 225 mg s.c. per month).

### Study procedures

Study procedures up to the end of the medication pause were described in detail elsewhere [17]. In brief, we collected headache data for the following time points: 1) four weeks prior to the first mAb treatment (baseline); 2) four weeks prior to the last mAb injection before treatment discontinuation; 3) weeks 13–16 after the last mAb injection.

**Fig. 1:**
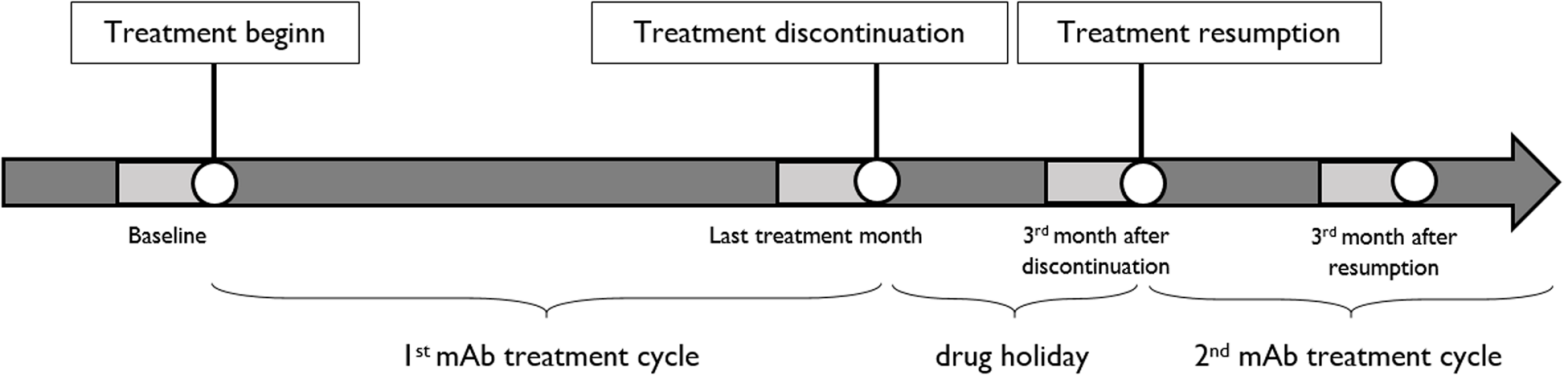
Study timeline. The periods marked in light grey correspond to the study observation periods

For this analysis, patients’ data were acquired from an additional visit 12 weeks after treatment reinitiation. Patients had to provide headache data for four consecutive weeks prior to this visit (weeks 9–12 after restart) (Fig. 1).

Headache data comprised monthly migraine days (MMD), monthly headache days (MHD) and monthly days with acute medication use (AMD). A migraine day was defined as any calendar day fulfilling the ICHD-3 criteria of a definite or probable migraine. We considered both triptans and non-specific pain medication (e.g. nonsteroidal anti-inflammatory drugs) as acute medication.

Patients with an improvement in MMD of ≥ 30% after treatment restart were considered responders, patients with < 30% improvement non-responders to the second treatment cycle.

At each visit except baseline, patients also completed the Headache Impact Test-6 (HIT-6). The HIT-6 is a validated questionnaire to assess the impact of headache on everyday life [18]. The achievable sum scores reach between 36 (no impairment) and 78 (very severe impairment).

### Outcomes

The primary endpoint of the study was the change in MMD between the last four weeks of treatment discontinuation and weeks 9–12 after restart. Secondary endpoints were the changes in MMD across the other observation points and the changes in MHD, AMD, and HIT-6 sum scores. The changes in headache parameters and HIT-6 scores in the receptor and ligand group were considered exploratory outcomes.

Further exploratory outcomes were the changes in headache parameters in responders and non-responders as well as patients with episodic and chronic migraine separately.

### Statistical analysis

Statistical analyses were performed using SPSS 27 (IBM, NY, USA). We summarized demographic data using descriptive statistics (mean ± standard deviation for numeric variables and absolute frequencies and percentages for categorical variables).

Since all outcomes of interest (MMD, MHD, AMD, HIT-6 scores) were not normally distributed, we used the Friedman test with post-hoc pairwise comparisons to assess primary and secondary endpoints. A two-tailed *p* value < 0.05 was considered statistically significant. *P* values were adjusted for multiple comparisons using the Bonferroni method. For exploratory endpoints we provided only descriptive values.

## Results

### Demographics and patients’ characteristics

The cohort consisted of *n* = 39 patients (*n* = 16 with erenumab, *n* = 15 with galcanezumab and *n* = 8 with fremanezumab, Table 1). The other *n* = 23 patients from the parent study [17] did not meet the inclusion criteria for this analysis: *n* = 8 restarted treatment after only one month of treatment discontinuation, *n* = 8 prolonged the treatment pause after three months, *n* = 7 switched treatment to another mAb class, i.e. from CGRP-R to CGRP mAb or vice versa.

**Table 1:** Demographic data and characteristics of the study cohort

**Variable**	**Full sample**	**Erenumab**	**Galcanezumab/ Fremanezumab**
**n**	39	16	23
**Sex (female)**	37 (94.9)	15 (93.8)	22 (95.7)
**Age (years)**	51.2 ± 11.1	52.3 ± 12.3	50.5 ± 10.4
**Chronic migraine**	25 (64.1)	10 (62.5)	15 (65.2)
**With aura**	20 (51.3)	8 (50.0)	12 (52.2)
**Months of treatment prior to discontinuation**	9.5 ± 1.0	9.9 ± 1.5	9.3 ± 0.4

Data is expressed as mean ± standard deviation or *n* (%).

### Migraine frequency before mAb treatment resumption

Patients reported 12.3 ± 5.4 MMD prior to the start of prophylaxis with a CGRP(-R) mAb. During therapy, MMD decreased to 7.5 ± 5.2 in the last treatment month. The discontinuation of mAbs led to an increase of MMD to 12.3 ± 6.3 at the end of the three-month discontinuation attempt similar to the data of the entire cohort [17].

By using CGRP(-R) mAbs, MHD decreased from 13.8 ± 6.4 to 7.7 ± 5.2 days. During the drug holiday, a MHD increase to 12.8 ± 6.1 was observed. In parallel, AMD were reduced from 11.0 ± 5.3 to 5.9 ± 4.6 under mAb treatment and increased again to 9.5 ± 5.3 after treatment discontinuation.

### Evolution of headache after mAb treatment resumption

**Fig. 2:**
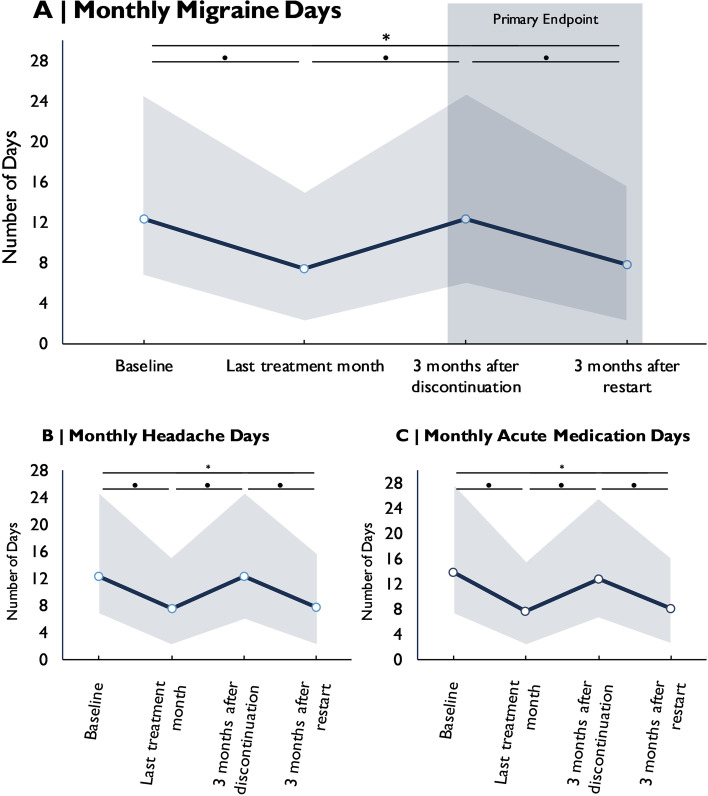
Migraine evolution before and after resumption of preventive treatment with CGRP(-receptor) mAbs. Evolution of monthly migraine days (**A**), monthly headache days (**B**), and monthly days with acute medication use (**C**) before the first mAb treatment cycle (baseline), at the end of the first treatment cycle, in the third month of treatment discontinuation and in the third month after restart. Values are mean ± standard deviation. ● = statistically significant between timepoints. * = statistically significant accross timepoints. Grey square = primary endpoint

The reinitiation of CGRP mAb therapy after the discontinuation period led to a significant reduction of MMD by -4.5 ± 4.9 after 9–12 weeks of therapy (*p* < 0.001) (Fig. 2A). MMD frequency returned to a level similar to the four-week period prior to the discontinuation attempt (*p* > 0.999).

MHD and AMD changed in a similar pattern with a significant improvement of frequency after treatment resumption (Fig. 2B and C). This trend was observed in both of the subgroups, i.e. patients treated with erenumab and patients on galcanezumab or fremanezumab (Table 2).

**Table 2:** Monthly migraine days, monthly headache days, and monthly days with acute medication use across the observation period in patients treated with the CGRP-R mAb erenumab and patients treated with the CGRP-mAbs galcanezumab or fremanezumab

**Monthly migraine days**				
	**Baseline**	**Last treatment month **	**3 months after discontinuation**	**3 months after restart**
**Erenumab**	12.9 ± 3.6	9.1 ± 5.7	14.5 ± 6.9	8.7 ± 6.1
**Galcanezumab/ Fremanezumab**	11.8 ± 6.4	6.4 ± 4.7	10.8 ± 5.5	7.1 ± 5.1
**Monthly headache days**				
	**Baseline**	**Last treatment month**	**3 months after discontinuation**	**3 months after restart**
**Erenumab**	14.9 ± 5.1	9.6 ± 5.6	15.2 ± 6.4	9.0 ± 5.8
**Galcanezumab/ Fremanezumab**	13.0 ± 7.2	6.4 ± 4.6	11.1 ± 5.3	7.4 ± 5.1
**Monthly days with acute medication**				
	**Baseline**	**Last treatment month**	**3 months after discontinuation**	**3 months after restart**
**Erenumab**	11.6 ± 4.2	6.6 ± 4.6	10.3 ± 6.2	5.1 ± 4.0
**Galcanezumab/ Fremanezumab**	10.7 ± 6.1	5.4 ± 4.7	9.0 ± 4.6	6.2 ± 5.1

Values are mean ± standard deviation (descriptive values, statistical significance is not provided)

### Responders vs. non-responders after treatment resumption

While *n* = 28 patients (72.8%) responded to the same mAb, in more than one-fourth of patients (*n* = 11, 28.2%) migraine frequency did not improve to a significant extent (> 30%) after mAb restart compared to the last month of the drug holiday. Patients were equally distributed between groups (erenumab *n *= 5, galcanezumab/fremanezumab *n* = 6). Demographic characteristics or headache data at baseline did not reveal any significant difference between non-responders and the remaining cohort (non-responders: *n* = 10, 90.9% women; *n* = 8, 72.7% with chronic migraine; 12.3 ± 5.7 MMD at baseline). However, the non-responders showed a significantly higher reduction of MMD during the first mAb treatment period than the responders did (3.6 ± 2.4 vs. 8.8 ± 5.3 MMD prior to medication pause, *p* = 0.003).

### Patients with episodic vs. chronic migraine after treatment resumption

In line with the entire cohort, patients with episodic and chronic migraine showed a worsening of MMD, MHD, and AMD to baseline levels after treatment discontinuation (Table 3). Three months after treatment resumption, headache parameters generally improved in both groups and returned to the levels of the last treatment month (Table 3). Of note, three patients with episodic migraine (21.4%) and eight patients with chronic migraine (32.0%) did not respond to the second treatment cycle.

**Table 3:** Monthly migraine days, monthly headache days, and monthly days with acute medication use across the observation period in patients with episodic migraine and patients with chronic migraine

**Patients with episodic migraine (*n*=14)**				
	**Baseline**	**Last treatment month **	**3 months after discontinuation**	**3 months after restart**
**Monthly migraine days**	8.4 ± 2.9	4.6 ± 3.6	7.8 ± 4.2	4.7 ± 3.3
**Monthly headache days**	8.9 ± 3.4	5.1 ± 4.2	8.4 ± 4.3	4.9 ± 3.2
**Monthly days with acute medication use**	7.3 ± 4.1	4.1 ± 3.4	6.6 ± 3.0	3.8 ± 3.0
**Patients with chronic migraine (*n*=25)**				
	**Baseline**	**Last treatment month **	**3 months after discontinuation**	**3 months after restart**
**Monthly migraine days**	14.4 ± 5.3	9.1 ± 5.4	14.8 ± 5.8	9.5 ± 5.8
**Monthly headache days**	16.6 ± 6.0	9.1 ± 5.3	15.2 ± 5.5	9.8 ± 5.6
**Monthly days with acute medication use**	13.1 ± 5.3	6.9 ± 5.0	11.3 ± 5.5	6.8 ± 5.1

Values are mean ± standard deviation (descriptive values, statistical significance is not provided)

### Changes in headache impact (HIT-6)

The HIT-6 sum scores decreased from 63.9 ± 4.5 in the third month of the drug holiday to 57.9 ± 5.6 in weeks 9–12 after treatment restart (*p* < 0.001), indicating a significant reduction of headache impact on everyday life. The HIT-6 scores after treatment reinitiation were very similar to those during the initial treatment cycle (58.9 ± 6.8, *p *> 0.999).

**Fig. 3:**
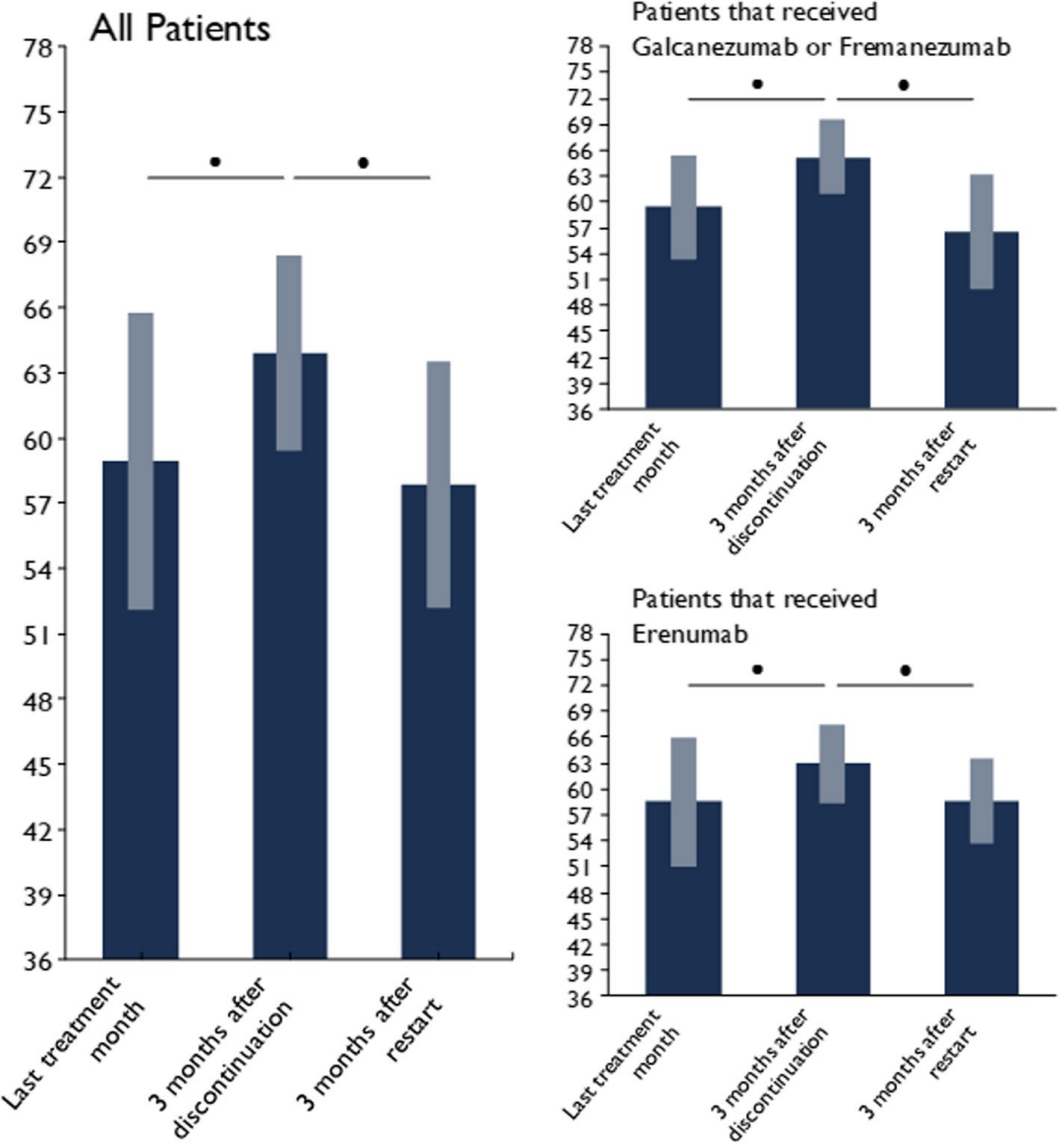
Mean HIT-6 sum scores before, during and after treatment discontinuation. Values are mean ± standard deviation. ● = statistically significant

In a subgroup analysis, patients with erenumab and patients with galcanezumab/fremanezumab showed a similar pattern (Fig. 3).

## Discussion

The majority of patients with migraine who resumed preventive treatment with the same CGRP(-R) mAb after a three-month drug holiday experienced a significant reduction of migraine frequency and acute medication use. HIT-6 scores improved, indicating a reduction of headache impact on everyday life. Migraine frequency after three months of mAb therapy following the drug holiday was similar to the frequency that was reported at the end of the first treatment period.

This data is relevant for clinical practice as most patients treated with a CGRP(-R) mAb in Europe undergo a drug holiday after 6–12 months as recommended by national and international guidelines, or even forced by the healthcare systems of some countries [14, 15, 19]. Recent studies have focused on migraine progression after treatment discontinuation. Patients with episodic migraine who received galcanezumab for six months in the EVOLVE-1 and -2 trials showed a progressive deterioration of migraine frequency within four months after treatment cessation but did not return to pretreatment baseline levels [20]. Worsening of migraine in a real-world setting appears more pronounced. De Matteis et al. reported a significant deterioration of migraine frequency as early as in weeks 1–4 after erenumab discontinuation [16]. In the study by Gantenbein et al., half of patients reached after three months of treatment pause a number of MMD comparable to the baseline phase [15]. Vernieri et al. also reported a gradual increase of MMD following cessation of treatment with erenumab and galcanezumab: After three months of drug holiday the 50% responder rates decreased from > 70% to < 30% [19]. Similarly, in the parent study of this analysis, we showed a significant increase of migraine frequency over time after CGRP(-R) mAbs treatment cessation [17], which is also observed in this subgroup analysis.

Headache data after a mAb drug holiday is only available for two small Italian cohorts: In the study by De Matteis et al., *n*  = 10 patients restarted erenumab treatment after only one month of treatment pause and showed a significant improvement of MMD and AMD in the first month after restart [16]. In the study by Iannone et al., *n* = 32 patients reported significantly improved MMD, AMD and HIT-6 scores in the first month of retreatment with erenumab or galcanezumab compared to the third month of treatment discontinuation [21]. Our results confirmed these preliminary findings and expand them to a longer time period.

Our findings are reassuring for patients who are advised to stop treatment with CGRP(-R) mAbs. The confirmation that almost 75% of patients have a favorable response after treatment resumption with the same mAb medication might reduce the fear of a drug holiday.

A lack of improvement after a drug holiday was described for other diseases. Multiple reports described a decreased effectiveness of lithium in patients with bipolar disorders after a treatment interruption, a phenomenon called “lithium-discontinuation-induced refractoriness” [22–25]. A possible neurobiological explanation is based on the phenomenon of episode sensitization: New and more severe episodes occurring during the drug holiday may cause neurobiological alterations resulting in a greater likelihood of recurrence [26]. This observation may apply also for some patients with migraine. Of note, about 25% of patients in this analysis did not benefit from treatment resumption with the mAb they previously responded to. These patients responded particularly well to the first treatment with mAbs prior to the drug holiday. The cause for the poorer response during the second treatment period remains to be determined. Anti-drug antibodies (ADA) may play a role in these patients. So far, ADAs against CGRP(-R) mAbs did not have any impact on their efficacy in randomized-controlled trials [27] albeit in a scenario without treatment interruption. The development of ADAs after treatment interruption or treatment restart has not been investigated yet.

The general recommendation of a drug holiday is based on the treatment with oral migraine preventatives. Oral drugs are commonly discontinued due to lack of efficacy or side effects, only 20% of patients continue treatment for one year [28]. Data from insurance companies’ databases reveals that only 10% of patients restart treatment with the same oral preventive drug after a discontinuation attempt [29]. As opposed to oral medications, a large proportion of our patients wanted to start again with the same mAb medication after three months at the latest [17]. Moreover, mAbs long-term data over several years demonstrated a consistent good efficacy and tolerability [30]. Given the recent evidence on migraine deterioration during discontinuation attempts [15–17, 19], the need for periodical treatment interruptions remains a matter of discussion.

This is the longest prospective analysis on treatment resumption with CGRP(-R) mAbs after a drug holiday. The inclusion and exclusion criteria allow for a very homogenous population. The small sample size constitutes a limitation and enables only exploratory analyses for the subgroups of patients with CGRP and CGRP-R mAbs. Given the real-world character of our investigation, we cannot control for placebo or nocebo effects during discontinuation or after restart.

## Conclusions

In conclusion, we showed a significant reduction of migraine frequency after re-initiation of treatment with CGRP(-R) mAbs. While over 70% of patients returned to the same migraine frequency comparable to the end of the first treatment cycle, in 30% of patients the response was not sufficient. Further research should aim to better characterize these patients and design personalized treatment regimens.

## Data Availability

The datasets used and/or analyzed during the current study are available from the corresponding author on reasonable request.

## Abbreviations

AMD: Monthly days with acute medication use
CGRP: Calcitonin Gene-Related Peptide
CGRP-R: Calcitonin Gene-Related Peptide-receptor
HIT-6: Headache Impact Test-6
ICHD-3: International Classification of Headache Disorders 3
mAb: Monoclonal antibody
MHD: Monthly headache days
MMD: Monthly migraine days
